# Temporal Patterns of Ant Diversity across a Mountain with Climatically Contrasting Aspects in the Tropics of Africa

**DOI:** 10.1371/journal.pone.0122035

**Published:** 2015-03-16

**Authors:** Thinandavha Caswell Munyai, Stefan Hendrik Foord

**Affiliations:** 1 Centre for Invasion Biology and Department of Ecology and Resource Management, University of Venda, Thohoyandou, Limpopo Province, South Africa; 2 Centre for Invasion Biology, South African Research Chair on Biodiversity Value & Change, and Department of Zoology, University of Venda, Thohoyandou, Limpopo Province, South Africa; Field Museum of Natural History, UNITED STATES

## Abstract

Factors that drive species richness over space and time are still poorly understood and are often context specific. Identifying these drivers for ant diversity has become particularly relevant within the context of contemporary global change events. We report on a long-term bi-annual (wet and dry seasons), standardized sampling of epigeal ants over a five year period on the mesic and arid aspects of an inselberg (Soutpansberg Mountain Range) in the tropics of Africa. We detail seasonal, annual and long-term trends of species density, test the relative contribution of geometric constraints, energy, available area, climate, local environmental variables, time, and space in explaining ant species density patterns through Generalized Linear Mixed Models (GLMM) where replicates were included as random factors to account for temporal pseudo-replication. Seasonal patterns were very variable and we found evidence of decreased seasonal variation in species density with increased elevation. The extent and significance of a decrease in species density with increased elevation varied with season. Annual patterns point to an increase in ant diversity over time. Ant density patterns were positively correlated with mean monthly temperature but geometric constraints dominated model performance while soil characteristics were minor correlates. These drivers and correlates accounted for all the spatio-temporal variability in the database. Ant diversity was therefore mainly determined by geometric constraints and temperature while soil characteristics (clay and carbon content) accounted for smaller but significant amounts of variation. This study documents the role of season, elevation and their interaction in affecting ant species densities while highlighting the importance of neutral processes and temperature in driving these patterns.

## Introduction

Understanding the overall importance of processes and correlates that determine diversity patterns has been an on-going challenge to biologists [1] as they act at different scales and require consideration of the taxa involved [2]. More than 30 hypotheses have been developed and tested that explain patterns of species richness along environmental and geographic gradients [3]. As a first approximation these hypotheses have been grouped into three categories, viz. null models, historical and ecological hypotheses [4, 5]. Recent studies have focused on a smaller number of these hypotheses [6]. Among these, the mid-domain effect [7], available area [8, 9], species energy-theory [10], soil properties [11] and habitat structure have been tested for many taxa (mammals [12, 13], birds [14], plants [15, 16] and insects [11, 17]).

The importance of understanding the impact of global change on biodiversity has brought renewed focus on mountains as living laboratories [18]. Their role in the study of richness patterns might also provide a predictive framework for the response of diversity to climate change [19]. An understanding of drivers of diversity along these gradients also identifies useful environmental filters for conservation initiatives [20]. Macro-ecological studies of species richness in mountains have documented the role of the mid-domain effect [21], temperature [22, 23], energy, and available area [24]. However, only a handful of studies have investigated temporal dynamics of diversity and very few have done so for more than a year, but see Bishop, Robertson [23].

The potential of mountains as replicated tests of the generality of these drivers [18] prompted the initiation of standardized long-term monitoring sites across three mountains in the major biomes of South Africa. These are the Cederberg mountains in the Cape Floristic Kindom [11], the Drakensberg mountains in the grassland biome [23] and the Soutpansberg mountain range in the savannah biome [39]. The Soutpansberg mountain is an inselberg in the north-eastern corner of South Africa. It lies north of the tropic of Capricorn and its eroded surface, varied topography, climate and erosion resistant quarzitic rock on diabase intrusions, dated at two billion years, have acted as a refuge and evolutionary hub for several endemic species [25–28]. The climate of the mountain is strongly influenced by its East North East to West South West orientation [29] resulting in an arid northern aspect characterized by open dry savannah and a mesic southern aspect with thicket, sedgeland/herbland, forest, and thicket/bushland habitats.

The role of ants in ecosystem dynamics has become increasingly important as the Anthropocene progresses [30] and developing an understanding of the factors that affect their diversity are both timely and relevant. Ant richness studies on mountains have reported a mid-elevation peak [21, 23, 31–34], monotonic decrease with increased elevations [32, 35, 36] or no clear pattern at all [11]. Most of the studies that measured and identified drivers and correlates of these patterns found temperature to be a significant factor while precipitation had a limited effect. Drivers such as available area played a minor role in two of the studies [22], whereas geometric constraints (mid-domain effect) was only relevant in one study [21]. These studies were done over short (< 1 year) temporal scales and assumes that patterns are constant in time and of lesser significance than spatial patterns [37]. Recent work has however focused on temporal variability over seasons [38] and among years [23]. Long-term sampling designs have observed new phenomena along elevations and allows for the investigation of hypotheses that cannot be tested with spatial designs only [23].

In a study of ants across the Soutpansberg, Munyai and Foord [39] identified a peak at mid-elevation on the arid northern aspect and a more complex pattern on the southern mesic aspect but did not consider temporal dynamics of diversity. This study aims to test whether there is any seasonal, annual and long-term trends of species density over a five year period (2009–2014) along this transect representing one of the longest time series of standardized sampling available for ants in the world and certainly the only one that falls within tropical regions of Africa. We test for a decrease in the seasonal variation in species density with increased altitude, as documented in a sister project [23] and we contrast the importance of correlates (soil, energy, habitat structure) and drivers (temperature, area and mid-domain effect) in explaining variation in ant richness with that explained by spatial and temporal predictors.

## Material and Methods

### Ethic Statement

The necessary permits for the described field study were obtained from Lajuma Research Centre and Goro Nature Reserve. This field study did not involve endangered or protected species.

### Study site

This study was carried out along a transect that extends over the highest point of the Soutpansberg mountain range (1748 m). The significance of this region has recently been affirmed by its inclusion into the UNESCO Man and Biosphere Program (MaB). The Soutpansberg is the main geographic feature within the Vhembe Biosphere Reserve (VBR), and include several core conservation areas critical to biodiversity conservation.

The transect was set out at 200 m elevation intervals on both sides (arid north and mesic south) of the mountain, with an altitudinal range of 900 m in the north (800–1700 m a.s.l.), and 800 m (900–1700 m) in the south. The gradient includes 11 elevational zones and is ca. 16 km in length. It stretches over a variety of habitats, viz. thicket/shrubland (900 m a.s.l. (9S); 1000 m a.s.l. (10S)), forests 1200 m a.s.l. (12S, tall forest); 1200 m a.s.l. (12S2, short forest)), sedgeland/herbland (1700 m a.s.l. (17N/summit); 1600 m a.s.l. (16S); 1400 m a.s.l. (14S)) in the south, open woodland (800 m a.s.l. (08N); 1000 m a.s.l. (10N); 1200 m a.s.l. (12N); 1400 m a.s.l. (14N)) in the north see [39] and [40] for further details.

### Ant Sampling

Sampling units consisted of 10 pitfall traps, each 62 mm in diameter, laid out in a grid (2 x 5) with 10 m spacing between traps. Sampling units within sites were replicated four times for each of the 11 elevational sites (44 sampling units in total). Replicates (sampling units) within an elevational zone were > 300 m apart to avoid pseudo-replication [39]. Pitfall traps were left open for five days each during September 2009, 2010, 2011, 2012 and 2013 (dry season), and January 2010, 2011, 2012, 2013 and 2014 (wet season) and contained 50% solution of propylene glycol that neither attract nor repel ants [41, 42]. The ant samples were washed in the laboratory and stored in 70% ethanol. They were sorted to morpho-species and where possible identified to species level.

### Correlates and drivers

#### Soil

Soil samples were collected in January 2010 using a soil auger. A total of ten soil subsamples in each of the 4 replicates of a site were pooled. Soils were dried and then analysed for composition: pH, conductivity, stone volume, K, Na, Ca, and Mg, C, NO3-N, H+, C, T-value, pBrayll, and the percentage clay, silt and sand by BemLab (pty) Ltd laboratories, Somerset West, South Africa (S1 Table). All 16 variables were standardized and principle component analysis performed to deal with co-linearity. The first four components explained 83% of the variation. Principal component axes one (Soil-PC1) and two (Soil-PC2) both represents a gradient from sandy to clayish soil, Soil-PC3 is associated with increased carbon content of the soil, and Soil-PC4 is linked to increased phosphate and sodium (S1 Fig.). Soil characteristics was only sampled during one sampling period and were therefore assumed to remain stable over the period of the study. Although this might not be true for many of the variables, the relative percentage of sand and clay that represents most of the variation between sites (Soil-PC1 and Soil-PC2) can be expected to remain relatively stable over a five year period.

#### Energy

Energy which is retained in the biomass produced by plants is available to consumers in the form of chemical energy [43]. Plant productivity is considered an appropriate measure of available energy [44]. Normalised difference vegetation index (NDVI) was derived for all sites during the sampling month i.e. from September 2009 to January 2014 (S7 Fig.). NDVI values were derived from MODIS (Moderate Resolution Imaging Spectroradiometer) /TerraVegetationIndicesMonthlyL3Global0.05DegCMGV005<https://lpdaac.usgs.gov/lpdaac/products/modis_product_table/vegetation_indices/monthly_l3_global_0_05deg_cmg/v5/terra > [44].

#### Habitat structure

Fine scale vertical and horizontal habitat structure were quantified during each of the 10 surveys [39]. The horizontal distribution of vegetation was determined by visually estimating percentage area covered by vegetation, leaf litter, exposed rock and bare ground on a 1 m² grid which was placed over each pitfall trap. For the vertical distribution of vegetation, the number of hits on a 1.5 m rod (i.e. the number of contacts with vegetation) was recorded at 25 cm intervals at four points located at 90° angles from a 1.5-m radius centred on each pitfall trap. An assessment of whether the rod would touch any vegetation anywhere above the rod (1.5 m) was also made. This provides some measure of canopy cover. Principle component analysis was performed on the horizontal and vertical measures respectively to account for co-linearity. For vertical habitat structure, each of the seven intervals along the rod was used as input variables. The PCA therefore not only accounts for the number of hits but also represents the location of the hits. The first two principal components accounted for 78% of the variation. Principal component axis one (Vert-PC1) represents a gradient from sites with dense canopy cover and very little ground cover to sites with low ground cover and the absence of canopy cover while Vert-PC2 is negatively related to increased complexity in vegetation structure (S5 Fig.). The first two principle components of horizontal habitat structure explained 81% of the total variation. Axis one (Hor-PC1) summarizes a gradient from sites dominated by bare ground and leaf litter to sites covered with vegetation, whereas axis two (Hor-PC2) contrast sites with rock cover with those dominated by leaf litter (S6 Fig.).

#### Temperature

Two Thermocron iButtons (Semiconductor Corporation, Dallas /Maxin, TX and USA) record soil temperature at 1 hour intervals at each site over the period of the study. The iButtons were buried 1 cm below the soil surface at locations that has direct exposure to sunlight except where canopy cover was > 70%. Temperature sequences from January 2009 to January 2014 were plotted and inspected for iButtons that malfunctioned or became exposed (S2 Fig.). The monthly mean, minimum, maximum temperature and variation in temperature (SD) at each site during the month of sampling were calculated (S3 and S4 Figs.). Temperature data for the two iButtons were averaged for each elevational site.

#### Area

The area covered by each elevation zone (200 m contour sampling zone) was calculated by creating a Minimum Convex Polygon of all replicate points with a 40 km buffer which encompasses the area under study, see Munyai and Foord [39] for further details.

#### Mid-domain effect

The eroded peneplain surrounding the Soutpansberg inselberg represents a hard boundary at lower elevations and the predictions of a null model was generated in RangeModel 5 [45] with similar protocols employed by Dunn, Colwell [46]. The boundaries were defined by the base of the mountain and the summit, i.e. model predictions included both the southern and northern aspects respectively. A species’ range was measured as the number of bins (bin = 200 m elevation intervals) between the highest and the lowest elevation site at which a species occurs. Occupancy was measured as the total number of bins at which a species occurs [46]. This was done for each of the aspects respectively.

### Spatio-temporal variables

Season (dry and wet) were included as categorical variables, year as numerical, aspect as a categorical variable, while spatial structures in the data were identified and modelled using principal coordinates of neighbour matrices (PCNM) by computing principle coordinates analysis (PCoA) of a truncated euclidean distance matrix that only have the distances between replicates that are close neighbours [47] (S8 Fig.) with the ‘stats’ package in R [48]. This is an eigenvector-based approach that allows for the modelling of spatial structures as predictor variables of variation in species density from broad to fine scales [49]. This is done by constructing spatial variables of all structures at relevant scales. The largest truncation distance determines the smallest scale perceived and large distances means the loss of smaller spatial scales. Four additional points (S2 Table) were therefore added to this design to reduce the threshold value, the PCNM values were calculated and the additional points removed from the PCNM matrix [50]. Thirty one eigenvectors (spatial variables) with significant positive spatial correlations were extracted and included as spatial variables for further analysis in the final model. This approach has several benefits as the size of eigenvalues are related to the scale it represents, spatial variables are orthogonal, represents a wide range of spatial scales and can model any type of spatial structure [50].

### Statistical analysis

Sample coverage works on the principle that samples of equal completeness and not equal size should be compared between communities. Here, sample completeness, was estimated with coverage-based rarefaction and prediction [51].

Variation in species density was analysed at three levels. We first modelled species density in response to correlates (soil) and drivers (habitat structure, temperature, energy and mid-domain). The second analysis modelled the ability of spatial (elevation and the eigenvectors of the principal components of neighbour matrices (PCNM’s) and temporal (season and year) to explain patterns in species density. This analysis also allowed us to determine if there are any intra-annual (season) and inter-annual (longer-term) trends ant species densities. These spatio-temporal predictors were then used to analyse the residuals of the first (environmental) model. This provides a measure of the variation in species density that is explained by pure spatial and temporal predictors [23].

Analysis was done with Generalized Linear Mixed Models (GLMM) using a loglink function and poisson error distribution. Replicates were included as random factors in the model to account for temporal pseudoreplication while all predictor variables were included as fixed variables. Observed species densities were weighted using sample coverage [52]. The Akaike Information Criterion (AICc) was used to discriminate between models and the best model was identified through manual backwards selection. Marginal R2 (R2m, due to fixed effects only) and conditional R2 (R2c, due to fixed and random effects) were calculated for the best model to determine how much of the variation is explained by fixed and random effects respectively [53].

The effect of elevation on seasonal variation was quantified by treating each replicate as a time series with ten data points from which the seasonal variation in species density was extracted with classical decomposition of moving averages [54], values were then regressed against elevation where a negative relationship indicates a decreased seasonal variation in species density at higher elevations. Species pool effects were corrected for by expressing species density as a proportion of the total number of species observed at the replicate over the period of the study and also as a proportion of the total number of species observed at a site over the period of the study, see [23].

## Results

**Fig 1:**
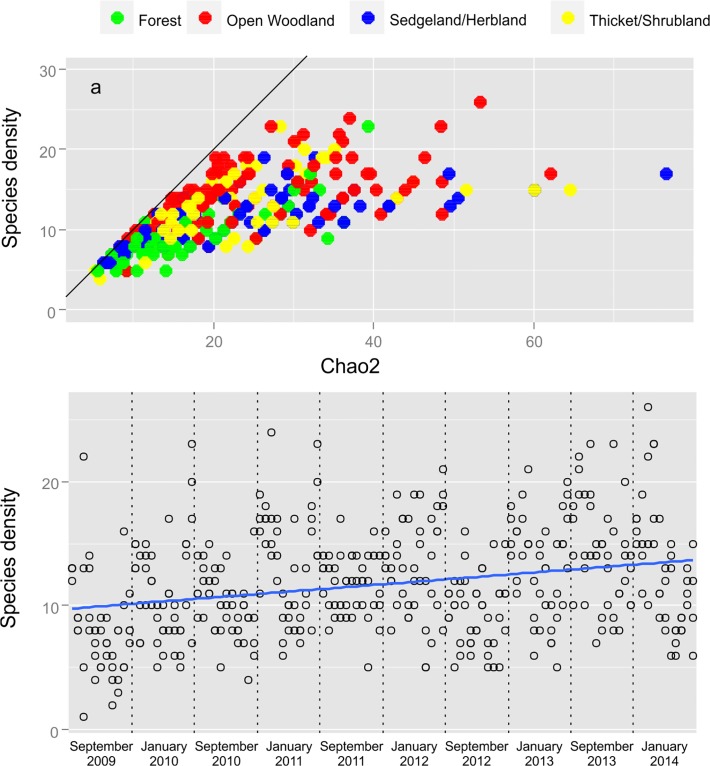
Scatterplots of Species Density Collected at 11 sites. a) Scatterplots of observed species densities and Chao 2 estimates of species richness at replicates in the four habitat types along the transect and b) a scatterplots of species densities collected at the 11 sites as a function of the chronological order of surveys.

**Fig 2:**
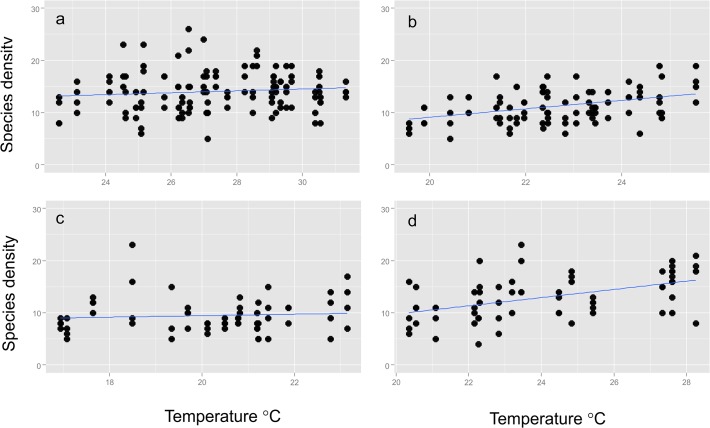
Scatterplots of Observed Species Density for Vegetation Types. Scatterplots of observed species densities against mean monthly temperatures recorded during the survey month when samples were collected in the a) open woodland, b) forests, c) sedgland/herbland, and d) thicket and shrubland.

A total of 86502 ants comprised of 130 species in 38 genera and six subfamilies were collected. Sample coverage for the whole survey varied from 0.98–0.99 for a site while the mean coverage was 0.82 ± 0.005 (SE) for replicates during a sampling occasion. Species densities observed in a replicate averaged at 15 species with the highest richness observed in the open woodlands followed by the thicket/bushland of the lower southern elevations and sedgeland/herblands of the higher elevations, while forest sites had the lowest observed species densities (Fig. 1). Scatterplots suggest that there is positive relationship between the average temperature during the month of the survey and the number of species observed (Fig. 2A—D). This pattern is the most evident for the thicket/shrubland and sedgeland/herbland habitats (Fig. 2C and D).

### Drivers and correlates

**Fig 3:**
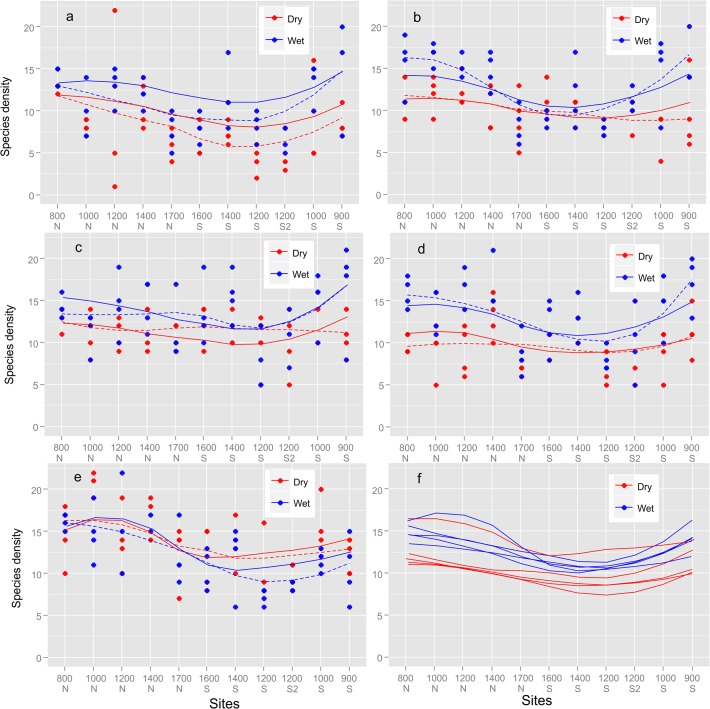
Species Density different Sampling Season. Species density for different sampling events (10 surveys) across the elevational sites in the Soutpansberg mountain range (2009–2014) predicted from the model, red lines (dry season) September survey and black lines (wet season) January surveys and also for all years (ten in total) combined. Also a plot for species density against all 10 surveys for all elevational sites from northern aspect to southern slope overtime (September 2009—January 2014), similarly top lines, red line = dry season and black lines = wet season.

The best model of species density retained average monthly temperature during the month of the survey, mid-domain effect, Soil-PC2, Soil-PC 3 and Soil-PC 4 as drivers and correlates (Table 1). The mid-domain effect’s estimate was highly significant (p < 0.001) and positive (Table 1). Average monthly temperature stood central to model performance and also performed the best when include as the only predictor in the model (Table 1). Average monthly temperature during the month of the survey also resembled the general form of ant richness patterns across the mountain (S2 Fig.). The best model had a marginal R2 of 0.33 while temperature alone explained 22% of the variation. Temperature’s estimate was positive (0.1 ± 0.02) suggesting that species density increases with increasing temperature. The model describes a pattern that is consistently higher along the lower elevations of the southern aspect, lower in the forests and summit and hump-shaped or unchanged on the northern aspect (Fig. 3). Sites of the open woodlands on the northern aspect generally had the highest observed and predicted species richness.

**Table 1:** Model AIC values, marginal and conditional R^2^ for generalized linear mixed models of all drivers, correlates, spatio-temporal and the residuals of the best driver model.

Drivers					
		ΔAIC (next best)	Worst model	R^2^_m_	R^2^_c_
Best Model	1450 (Average Temperature + Mid-domain + Soil-PC2 + Soil-PC3 + Soil-PC4)	1452	1463.7	0.33	0.38
Average Temperature	1497.073			0.22	0.32
Mid-domain	1484.767			0.16	0.37
Soil-PC2	1540.136			0.06	0.27
Soil-PC3	1544.527			0.03	0.27
Soil-PC4	1547.546			0.005	0.27
Spatio-temporal					
		ΔAIC (next best)	Worst model	R^2^_m_	R^2^_c_
Best model	1489.437 (Elevation:Season+Year + Season + PCNM2 + PCNM3 + PCNM11 + PCNM13 + PCNM14 + PCNM17 + PCNM25)	1490		0.32	0.32
Year	1532.8			0.06	0.3
Season	1529.6			0.05	0.3
PCNM2	1537			0.08	0.26
Residuals of drivers model				0.04	0.52

### Spatio-temporal covariates

Species density was higher during the wet seasons (Table 2) but there was an exception to this rule in the 2013/2014 sampling season when some sites on the southern aspect recorded higher species densities during the dry season than during the wet season (Fig. 3).The interaction of elevation with season was negative and significantly so for the wet season. This points to a more pronounced decrease in species density with elevation. There has also been a significant annual increase in species densities over the period of the study (Table 2). Seven of the 31 spatial eigenvectors (PCNM’s) were retained in the best model and their spatial structure is shown in S9 Fig. These seven variables represents spatial structuring of ant species density patterns from the largest to the smallest spatial scale.

**Table 2:** Estimates of significant terms in spatio-temporal and driver-correlate models of species densities observed over the period of the study.

Spatio-temporal	
Term	Estimates
Year	0.04 ± 0.015*
Dry Season ↔ Wet Season	0.4 ± 0.04 *
Elevation: Wet Season	−0.0004 ± 0.9 × 10^–4^ *
Elevation: Dry Season	−0.0001 ± 0.9 × 10^–4^
PCNM2	0.0001 ± 0.1 × 10^–4^ ***
PCNM3	−0.00004 ± 0.1 × 10^–4^ *
PCNM11	−0.00007 ± 0.3 × 10^–4^ *
PCNM13	0.0001 ± 0.4 × 10^–4^ **
PCNM14	−0.00007 ± 0.3 × 10^–4^ **
PCNM17	0.00009 ± 0.4 × 10^–4^ *
PCNM25	0.0006 ± 0.3 × 10^–4^.
Drivers and correlates	
Mid-domain	0.12 ± 0.02 ***
Monthly Average Temperature	0.1 ± 0.02 ***
Soil-PC2	0.48 ± 0.14 ***
Soil-PC3	−0.26 ± 0.15.
Soil-PC4	0.26 ± 0.14.

., < 0.01;

*, < 0.05

**, < 0.01

***, < 0.001

### Analysis of model residuals

Spatio-temporal variables explained a small amount of variation in the residuals of the best driver model, R2m = 0.04, and suggest that there were no spatio-temporal pattern in the residuals of the best driver model and that all the variation is explained by temperature and mid-domain effect.

### Seasonal variation in species density

**Fig 4:**
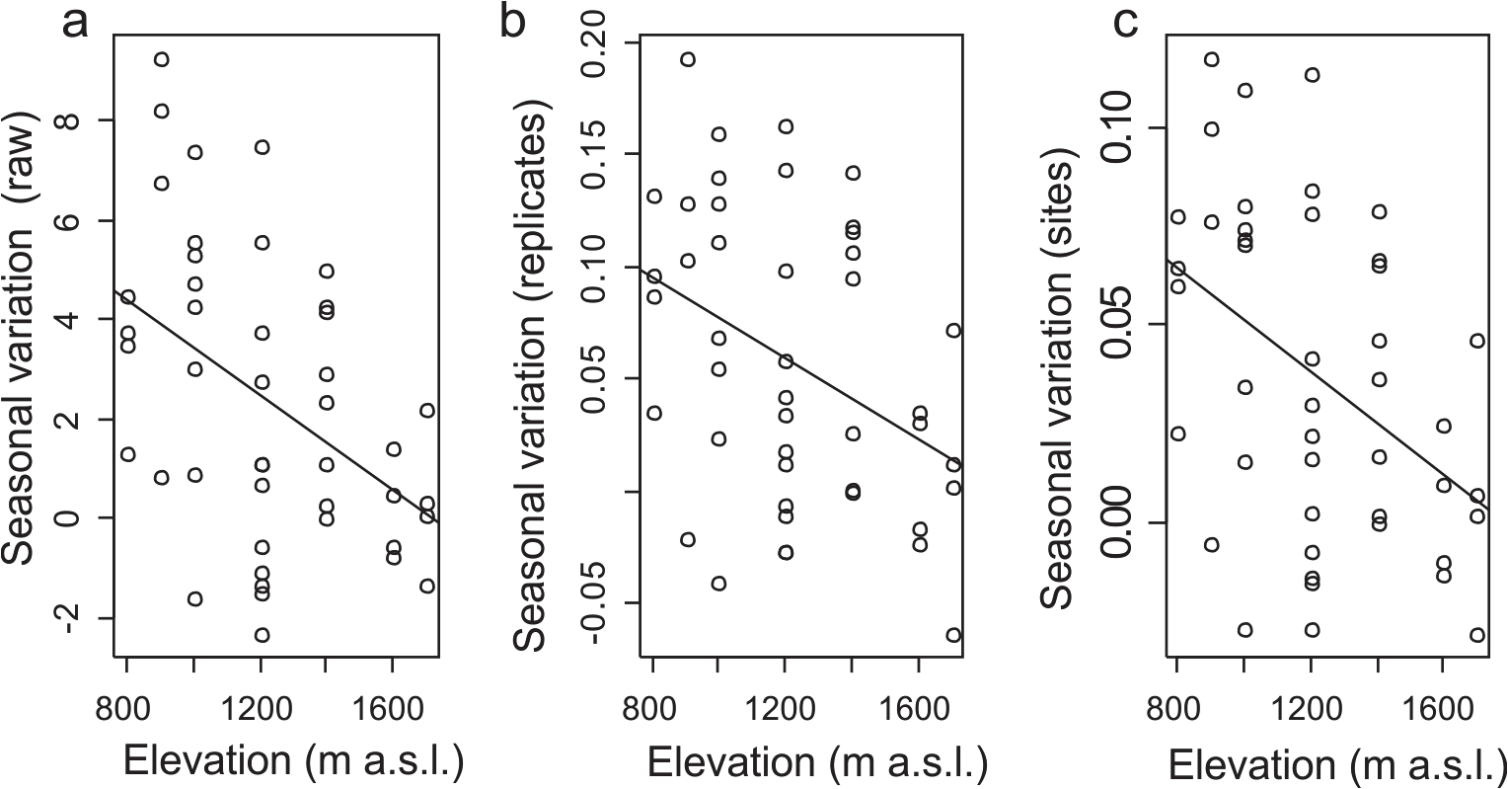
Seasonal Variation and Elevation. Relationship between seasonal variation in species density and elevation on both aspects of the mountain for a) raw species density, b) as proportion of total species observed in a replicate over the period of the study, c) and proportion of the total species observed at a site (elevational band).

Seasonal variation in species density decreased significantly (b = −0.5–2, df = 42, p = 0.002) with increased elevation even when the size of total species in replicates (b = −0.9–4, df = 42, p = 0.001) and sites (b = −0. 9–4, df = 42, p = 0.003) or elevational bands were accounted for (Fig. 4).

## Discussion

The most widely accepted pattern of richness along altitudinal gradients is the decline in richness with increased elevation [55] although there has been growing support of richness peaking at mid-elevation [21, 23, 31, 33, 34, 56, 57]. Richness patterns along this transect are more complex with diversity peaking at mid-elevation on the northern slope of the mountain, decreasing at higher elevations [35, 36, 58, 59] followed by a small intermediate peak towards mid-elevation before reaching a peak at the lowest elevation of the southern slope, see Fig. 3F. The patterns remained constant throughout the study period.

Ant species density was mostly higher during the wet seasons. This is not surprising as ants are most active and abundant during this period (summer rainfall periods) in southern Africa [60]. The reversal of this trend during the 2013/2014 sampling season, when species densities were higher during the dry season, point to the considerable heterogeneity and variability observed in this savannah biome compared to the grassland biome in Southern Africa where wet seasons were consistently more diverse than dry seasons and could explain why models in our study explained less variation than models for ant diversity along elevations in grassland [23]. After controlling for the size of species pools (Fig. 4), the seasonal variation in species density still decreased with elevation, strengthening support for the impact of elevation on seasonal effects [23]. Thus an increased richness was not the result of dispersal from the regional species pool but caused by decreased activity levels of ant colonies during cold and resource poor periods (dry season). These conditions have a disproportionately higher impact on lower elevation species. These taxa are often less tolerant of colder temperatures experienced during the dryer seasons. Many of the predatory specialist, several of which were restricted to lower elevations e.g. ponerinae ants [61] were also only active during the wet season when prey availability peaked. Ant species density showed a significant increase over the period of the study (Fig. 1B).

The mid-domain effect played a prominent role in explaining variation in species density. Evidence for the importance of mid-domain effect has been found in other studies Dunn et al. [42] but support also exists for the contrary [22, 62]. Sanders [21] in a regional elevational gradient study, reported on the importance of geometric constraints in explaining ant richness variation where the mid-domain effect together with available area explained 90%, 99% and 57% in Colorado, Nevada and Utah respectively. In contrast, a study in southern Appalanchians of leaf-litter ants along an altitudinal gradient found no support for geometric constraints models [22]. Kaspari, Ward [63] also found no support for these models at a continental scale in North America. While Bishop, Robertson [23], although not directly testing for these models, argued that it is unlikely that neutral models are responsible for driving patterns across their study area and pointed to the importance of temperature and area in explaining diversity patterns observed. Our results highlight the importance of geometric constraints and not area in explaining variation in richness.

There was a significant positive association between species density and percentage clay in the soil (Soil-PC2). Percentage clay negatively affected the dominance levels of dominant ants in the Kruger National Park [64] and distribution of Anoplolepis cf. custodiens in the Cederberg [11]. The abundance of the dominant ant Messor andrei, in North Californian grasslands, was negatively associated with percentage clay levels [65]. While it was positively related with the occurrence of more common ant species, Acropyga fuhrmanni in Ecuador [66]. In our study high percentage clay of the soil was associated with high species density and corresponds with Ramon, Barragan [67] observation that clay contribute to high species richness in Ecuadorian Andean forests. Although Delsinne, Roisin [68] found no relationship between soil texture and alpha diversity, their study further suggest the indirect effect of soil [66] on ant diversity through its impacts on plants e.g. excess salt in the soil negatively affect ant diversity through its effect on plant diversity.

At a larger and regional scale, Braschler, Chown [44] observed a unimodal relationship between ant richness and NDVI with a peak at average productivity in sites of the Fynbos biome. The smaller scale of this study might explain why energy failed to have an effect [69]. NDVI might also be a poor proxy of available energy in this study. Particularly for the forests and habitats with considerable canopy cover where very little of this biomass will be available for epigeal ants.

Studies along an arid elevational transect stressed the importance of temperature where in combination with precipitation explained 80% of ant species variation [32]. Temperature constrains ant foraging activities as it restricts their foraging times of the day [70]. It is also widely accepted as a principal factor that determines distribution and activities [71] and there is a growing evidence that low temperature is a primary stress that controls patterns in ants as well as community structure [72, 73]. In contrast a study in Indonesian cacao plantations by Wielgoss, Tscharntke [74] observed a negative correlation between temperature and ant species richness due to the presence of aggressive dominant Dolichoderinae ant species in the study area. Complex environments also have an influence on temperature indirectly, where open habitats tend to have high temperatures compared to closed/complex environment [72].

Similar to our study, complex habitats and low temperatures at higher elevations of Serra do Cipó [73] sampled less ants species. Soil temperatures in the thickets/shrubland and sedgeland/herbland habitats were a better predictor of species density than the open woodlands and forest. Thermal ranges in the forests are small (S3 Fig. (12S and 12S2)) and might act as a filter for a small subset of cold tolerant species. In contrast, open woodlands are characterised by considerable variation in micro-climates over very small scales. The two iButtons per site might therefore not capture the varied thermal regimes of this habitat and fail to capture some of the variation in observed species density.

In conclusion, we found that geometric constraints were the most important driver of species density patterns followed by temperature while soil characteristics played a minor role. Energy, habitat structure and area failed to explain significant amounts of variation while spatio-temporal predictors were accounted for by the best model of drivers. In contrast to our sister transect in the Drakensberg mountains [23], we’ve observed an increase in species density over time. Similar to their study, seasonal variation in species density decreased with elevation. There was a significant decrease in richness with elevation during the wet season while this decrease was not significant during the dry season. The strength of patterns therefore varied with season and elevation.

## Supporting Information

S1 Table Soil Properties. Soil properties of all 44 replicates along Soutpansberg transect.
(PDF)

S2 Table UTM Coordinates of Replicates. UTM coordinates of replicates as well as dummy variables that were included to reduce the threshold value and reduce the smallest scale that can be perceived.
(PDF)

S1 Fig Site and soil properties Bioplot. Site and soil properties biplot of Principal Component Analysis. Red arrows indicate soil properties and black labels indicate replicates.
(TIF)

S2 Fig Boxplot of soil temperature. Boxplot of soil temperature at each site over the period of the study.
(TIF)

S3 Fig Boxplot of Soil Temperature Monthly_a. Boxplot of soil temperatures at sites during the months when ants were sampled.
(TIF)

S4 Fig Boxplot of Soil Temperature Monthly_b. Boxplot of soil temperatures at sites during the months when ants were sampled.
(TIF)

S5 Fig Vertical Vegetation Structure. Vertical vegetation structure of all 44 replicates (averaged over the period of the study) along Soutpansberg transect.
(TIF)

S6 Fig Horizontal Vegetation Structure. Horizontal vegetation structure of all 44 replicates (averaged over the period of the study) along Soutpansberg transect.
(TIF)

S7 Fig Boxplot of NDVI. Boxplot of NDVI at each site over the period of the study.
(TIF)

S8 Fig UTM for 44 Replicates. UTM coordinates of 44 replicates along transect as well as the four dummy variables included in the Principal Coordinate Analysis of Neighbourhood Matrices. Inset is the minimum spanning tree used to calculate the maximum distance required to connect all replicates.
(TIF)

S9 Fig Representation of Significant. Representation of significant eigenvectors in geographical space.
(TIF)
