# Supplementary material for: Temporal Patterns of Ant Diversity across a Mountain with Climatically Contrasting Aspects in the Tropics of Africa
Source: PLoS One. 2015 Mar 16;10(3):e0122035. doi: 10.1371/journal.pone.0122035 (PMC4361397; doi:10.1371/journal.pone.0122035)
Supplement: S1 Table — Soil properties of all 44 replicates along Soutpansberg transect. (PDF) [file pone.0122035.s001.pdf]

eplicate	Soil	pH	Resist.	H^+	Stone	P Bray II	K
Site	Soil	(KCl)	(Ohm)	(cmol/kg)	(Vol %)	mg/kg	
8N1	Sand	4.2	14650	0.51	1	2	24
8N2	Sand	5.6	770	0.35	1	9	521
8N3	Sand	4.4	8270	0.71	1	7	52
8N4	Sand	4	12900	0.71	1	7	21
10N1	Sand	4.4	1240	0.76	1	18	68
10N2	Sand	3.5	1760	2.93	1	55	50
10N3	Sand	5.3	3120	0.76	1	25	249
10N4	Sand	3.9	15600	0.96	1	8	21
12N1	Sand	3.7	12860	1.36	1	6	21
12N2	Sand	3.8	11000	1.41	27	4	24
12N3	Sand	3.1	6800	6.62	1	8	51
12N4	Sand	3.6	7370	1.77	1	6	23
14N1	Sand	3.9	7450	1.57	8	11	26
14N2	Sand	3.9	14240	1.57	1	9	36
14N3	Sand	3.8	18240	1.06	1	9	15
14N4	Sand	4	8000	1.26	1	6	23
17N1	Sand	4.2	9050	2.02	1	10	52
17N2	Sand	4	5130	3.03	19	13	67
17N3	Sand	3.7	5050	4.7	1	46	176
17N4	Sand	4	13190	3.33	1	21	60
16S1	Sand	3.9	2650	5.61	1	14	56
16S2	Sand	4	3590	4.04	4	12	46
16S3	Sand	3.9	1440	5.5	16	26	82
16S4	Sand	4.1	8870	2.37	1	11	34
14S1	Sand	4.1	5480	1.41	4	12	47
14S2	Sand	4.2	8420	1.26	1	8	21
14S3	Sand	3.9	5970	1.77	1	32	22
14S4	Sand	3.9	5300	1.77	1	53	227
12S1	Sand	6	1130	0.35	1	56	359
12S2	Sand	5.1	1400	1.31	1	16	262
12S3	Sand	6.4	690		1	42	382
12S4	Sand	6.2	650		1	4	725
12S2.1	Sand	4.5	3170	0.86	1	4	142
12S2.2	Sand	4.5	1580	1.67	1	7	291
12S2.3	Sand	5.4	1000	0.86	1	5	196
12S2.4	Loam	5	2680	1.26	1	6	404
10S1	Sand	5.2	1260	0.86	1	4	119

Orchard	Soil	pH	Resist.	H^+	Stone	P Bray II	K
Site	Soil	(KCl)	(Ohm)	(cmol/kg)	(Vol %)	mg/kg	
10S2	Loam	5.8	1170	1.52	1	68	408
10S3	Sand	5.1	850	1.01	1	3	111
10S4	Loam	5.1	1400	0.86	1	3	117
9S1	Loam	4.8	970	1.36	1	4	186
9S2	Clay	5.1	820	0.86	1	3	183
9S3	Loam	4.5	2670	1.01	1	4	201
9S4	Sand	4.6	2940	0.96	1	3	269

Replicate	Exchangeable cations (cmol(+)/kg)				C	NO_3-N	Na	K
Site	Na	K	Ca	Mg	%	mg/kg	%	%
8N1	0	0.06	0.3	0.09	0.28	3.54	0	6.46
8N2	0.07	1.33	3.15	1.76	1.24	40.4	1.1	19.98
8N3	0	0.13	0.82	0.35	0.72	6.26	0	6.6
8N4	0	0.05	0.3	0.1	0.48	3.97	0	4.73
10N1	0.05	0.17	0.9	0.31	0.62	31.52	2.17	7.95
10N2	0.08	0.13	0.39	0.09	2.34	34.84	2.2	3.53
10N3	0.09	0.64	4.43	1.69	2.26	9.08	1.23	8.37
10N4	0.01	0.05	0.41	0.06	0.3	3.85	0.49	3.58
12N1	0	0.05	0.07	0.01	0.99	3.51	0	3.59
12N2	0	0.06	0.06	0.01	0.94	3.85	0	4
12N3	0.03	0.13	0.75	0.23	3.98	3.2	0.36	1.69
12N4	0	0.06	0.24	0.06	1.59	5.32	0	2.79
14N1	0.03	0.07	0.06	0.02	0.95	5.54	1.58	3.81
14N2	0.02	0.09	0	0.01	0.73	2.83	1.16	5.45
14N3	0.02	0.04	0	0	0.88	3.6	2.22	3.38
14N4	0	0.06	0.41	0.07	0.5	5.33	0	3.22
17N1	0.04	0.13	0.35	0.24	1.79	6.91	1.45	4.8
17N2	0.04	0.17	0.16	0.1	3.44	9.12	1.27	4.9
17N3	0.07	0.45	0.58	0.27	4.4	8.02	1.12	7.39
17N4	0.04	0.15	0.59	0.08	3.71	3.72	1.06	3.64
16S1	0.03	0.14	0.22	0.1	4.98	39.27	0.51	2.33
16S2	0.05	0.12	0.66	0.3	3.6	18.25	0.99	2.26
16S3	0.11	0.21	0.53	0.23	4.47	39.9	1.61	3.2
16S4	0.04	0.09	0.13	0.03	1.82	6.98	1.65	3.28
14S1	0.01	0.12	0.41	0.14	1.33	8.16	0.42	5.8
14S2	0.02	0.05	0.15	0.06	0.71	6.93	1.06	3.53

Replicate	Exchangeable cations (cmol(+)/kg)				C	NO_3-N	Na	K
Site	Na	K	Ca	Mg	%	mg/kg	%	%
14S3	0	0.06	0.4	0.24	1.16	6.84	0	2.32
14S4	0.03	0.58	0.2	0.16	1.15	8.65	0.97	21.26
12S1	0.04	0.92	11.12	3.11	2.53	22.73	0.28	5.9
12S2	0.16	0.67	11.33	3.88	5.06	2.25	0.95	3.87
12S3	0.14	0.98	18.51	4.69	5.11	78.86	0.56	4.02
12S4	0.11	1.85	11.55	2.93	2.34	54.48	0.65	11.27
12S2.1	0.04	0.36	1.9	0.79	0.69	11.61	1.02	9.18
12S2.2	0.08	0.75	4.75	2.12	2.42	20.18	0.86	7.95
12S2.3	0.13	0.5	13.33	7.43	5.14	21.43	0.58	2.26
12S2.4	0.12	1.03	9.71	4.04	2.3	34.99	0.75	6.39
10S1	0.14	0.3	8.54	7.56	3.33	6.71	0.81	1.75
10S2	0.09	1.04	9.02	2.78	1.84	13.9	0.63	7.22
10S3	0.15	0.28	9.11	5.39	2.31	21.15	0.97	1.78
10S4	0.1	0.3	7.5	5.09	1.61	10.41	0.72	2.17
9S1	0.12	0.48	10.44	5.82	2.01	11.26	0.65	2.61
9S2	0.09	0.47	10.73	7.19	1.66	14.11	0.47	2.42
9S3	0.08	0.51	4.5	2.89	0.25	4.11	0.86	5.72
9S4	0.06	0.69	3.38	1.86	1.72	3.88	0.93	9.87

Orchard	Ca	Mg	T-Value	Clay	Silt	Sand	Classification
Site	%	%	cmol/kg	%	%	%	Classification
8N1	31.2	9.69	0.97	0.8	0.2	99	Sa
8N2	47.29	26.38	6.66	0.6	0.8	98.6	Sa
8N3	40.89	17.31	2.02	0.8	0.2	99	Sa
8N4	25.64	8.49	1.16	1	0.4	98.6	Sa
10N1	41.11	14.08	2.19	0.6	1.8	97.6	Sa
10N2	10.87	2.48	3.62	1	0.4	98.6	Sa
10N3	58.19	22.23	7.62	1	0.4	98.6	Sa
10N4	27.56	4.09	1.49	1	0.4	98.6	Sa
12N1	4.54	0.69	1.49	0.6	0.8	98.6	Sa
12N2	3.97	0.86	1.55	0.8	0.2	99	Sa
12N3	9.67	2.94	7.76	1	0.4	98.6	Sa
12N4	11.35	2.97	2.14	0.6	0.8	98.6	Sa
14N1	3.56	1.37	1.75	3.4	0.4	96.2	Sa
14N2	0.12	0.45	1.69	3	2	95	Sa
14N3	0	0	1.12	0.6	0.8	98.6	Sa

Orchard	Ca	Mg	T-Value	Clay	Silt	Sand	Classification
Site	%	%	cmol/kg	%	%	%	Classification
14N4	22.88	4.08	1.8	0.4	1	98.6	Sa
17N1	12.47	8.55	2.78	3.4	0.4	96.2	Sa
17N2	4.44	2.74	3.5	3.4	0.2	96.4	Sa
17N3	9.57	4.53	6.07	3.6	0.2	96.2	Sa
17N4	14.03	2	4.2	3.4	1.4	95.2	Sa
16S1	3.57	1.59	6.1	3.4	0.2	96.4	Sa
16S2	12.75	5.83	5.17	3.4	0.2	96.4	Sa
16S3	8.13	3.45	6.58	3.4	0.2	96.4	Sa
16S4	4.75	1.17	2.66	3.4	0.2	96.4	Sa
14S1	19.73	6.54	2.09	3.2	0.2	96.6	Sa
14S2	9.86	3.62	1.54	3.2	0.2	96.6	Sa
14S3	16.19	9.61	2.46	3	2	95	Sa
14S4	7.24	5.7	2.73	3.2	2.2	94.6	Sa
12S1	71.57	19.99	15.54	3	1	96	Sa
12S2	65.3	22.34	17.35	3.2	0.2	96.6	Sa
12S3	76.11	19.31	24.31	3	3	94	Sa
12S4	70.24	17.84	16.45	1	6	93	Sa
12S2.1	48.04	20.01	3.95	2	5	93	Sa
12S2.2	50.75	22.61	9.37	1	3	96	Sa
12S2.3	59.9	33.39	22.25	2	4	94	Sa
12S2.4	60.05	25.02	16.16	0	9	91	Sa
10S1	49.07	43.43	17.4	3	6.4	90.6	Sa
10S2	62.43	19.2	14.45	13.2	8.2	78.6	SaLm
10S3	57.11	33.8	15.96	9.4	11.8	78.8	LmSa
10S4	54.14	36.77	13.85	11	13	76	SaLm
9S1	57.31	31.96	18.22	17.2	9.8	73	SaLm
9S2	55.46	37.2	19.34	21.4	9.8	68.8	SaKlLm
9S3	50.02	32.18	9	12	3.4	84.6	SaLm
9S4	48.61	26.78	6.96	9.6	1.8	88.6	LmSa
