# Supplementary figures and images for: Temporal Patterns of Ant Diversity across a Mountain with Climatically Contrasting Aspects in the Tropics of Africa
Source: PLoS One. 2015 Mar 16;10(3):e0122035. doi: 10.1371/journal.pone.0122035 (PMC4361397; doi:10.1371/journal.pone.0122035)

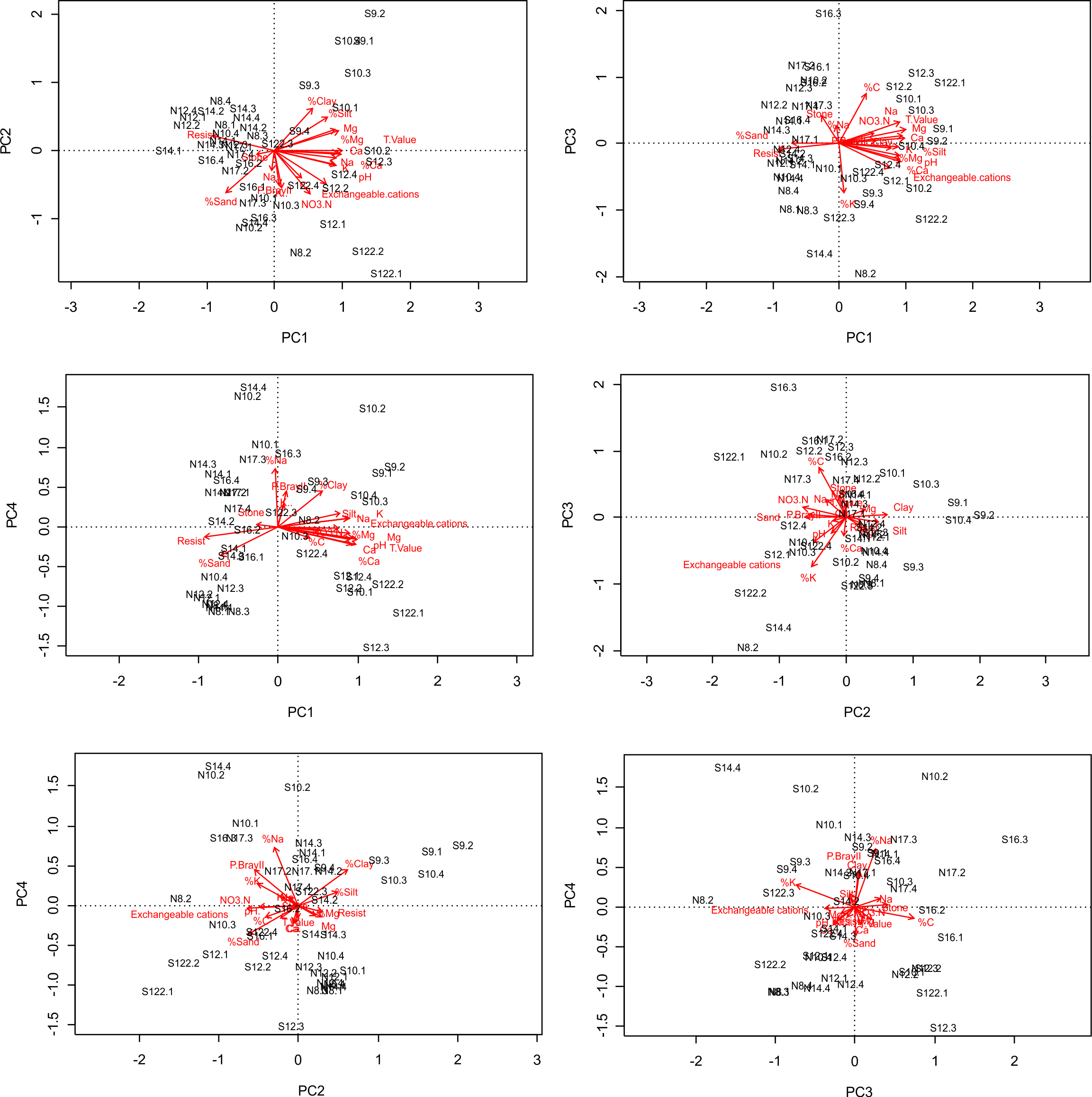

Supplement: S1 Fig — Site and soil properties biplot of Principal Component Analysis. Red arrows indicate soil properties and black labels indicate replicates. (TIF) [file pone.0122035.s003.tif]

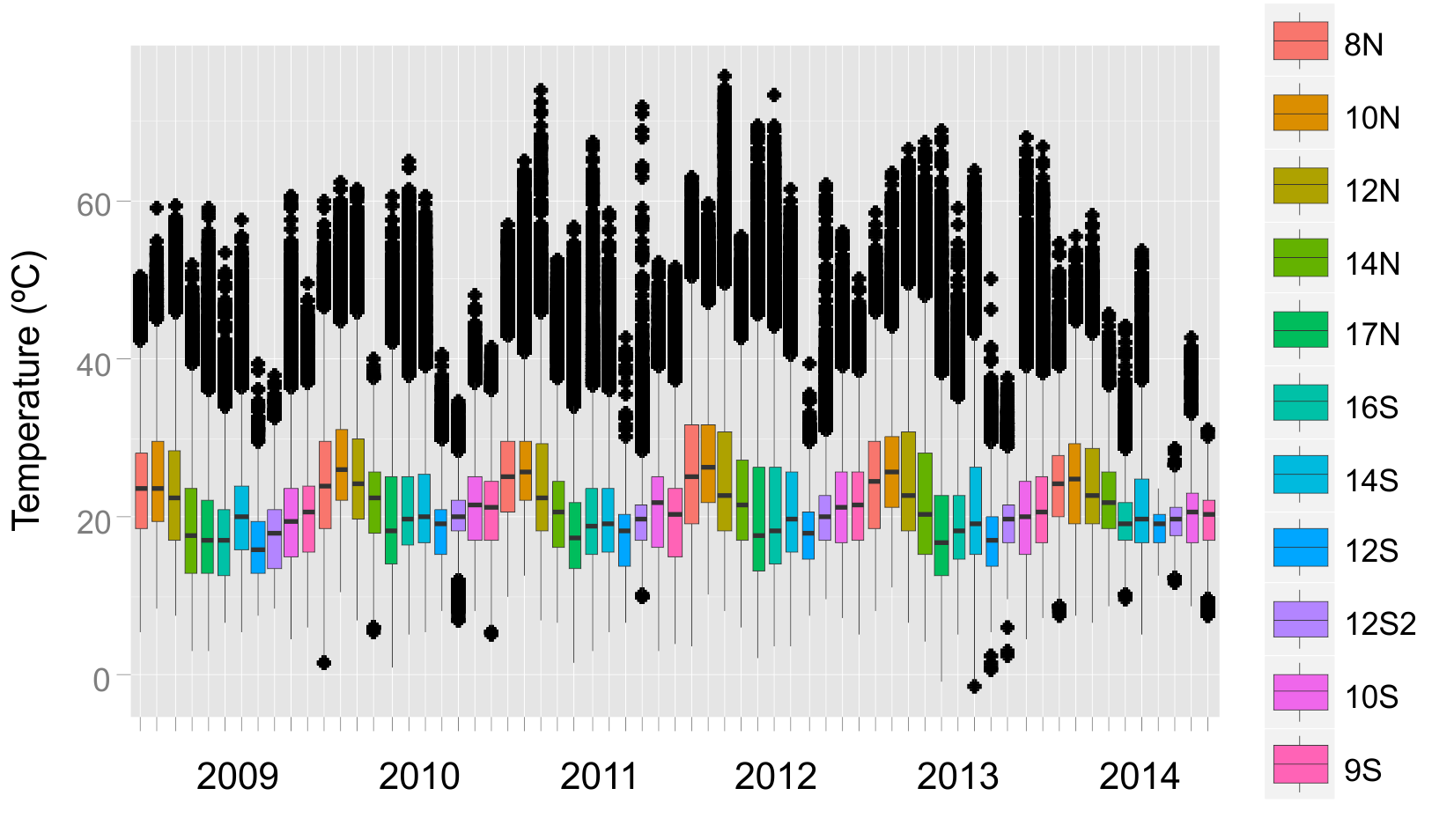

Supplement: S2 Fig — Boxplot of soil temperature at each site over the period of the study. (TIF) [file pone.0122035.s004.tif]

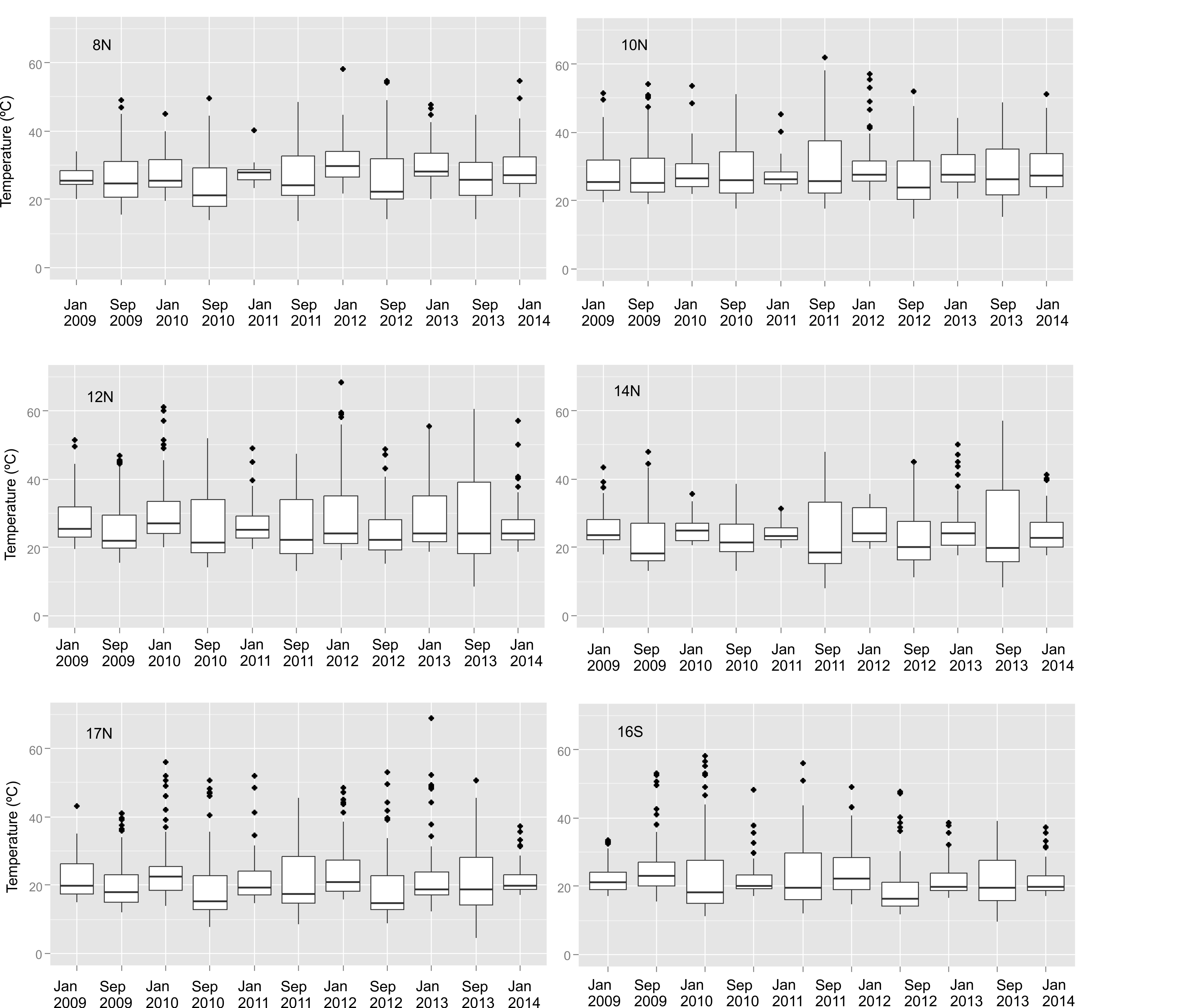

Supplement: S3 Fig — Boxplot of soil temperatures at sites during the months when ants were sampled. (TIF) [file pone.0122035.s005.tif]

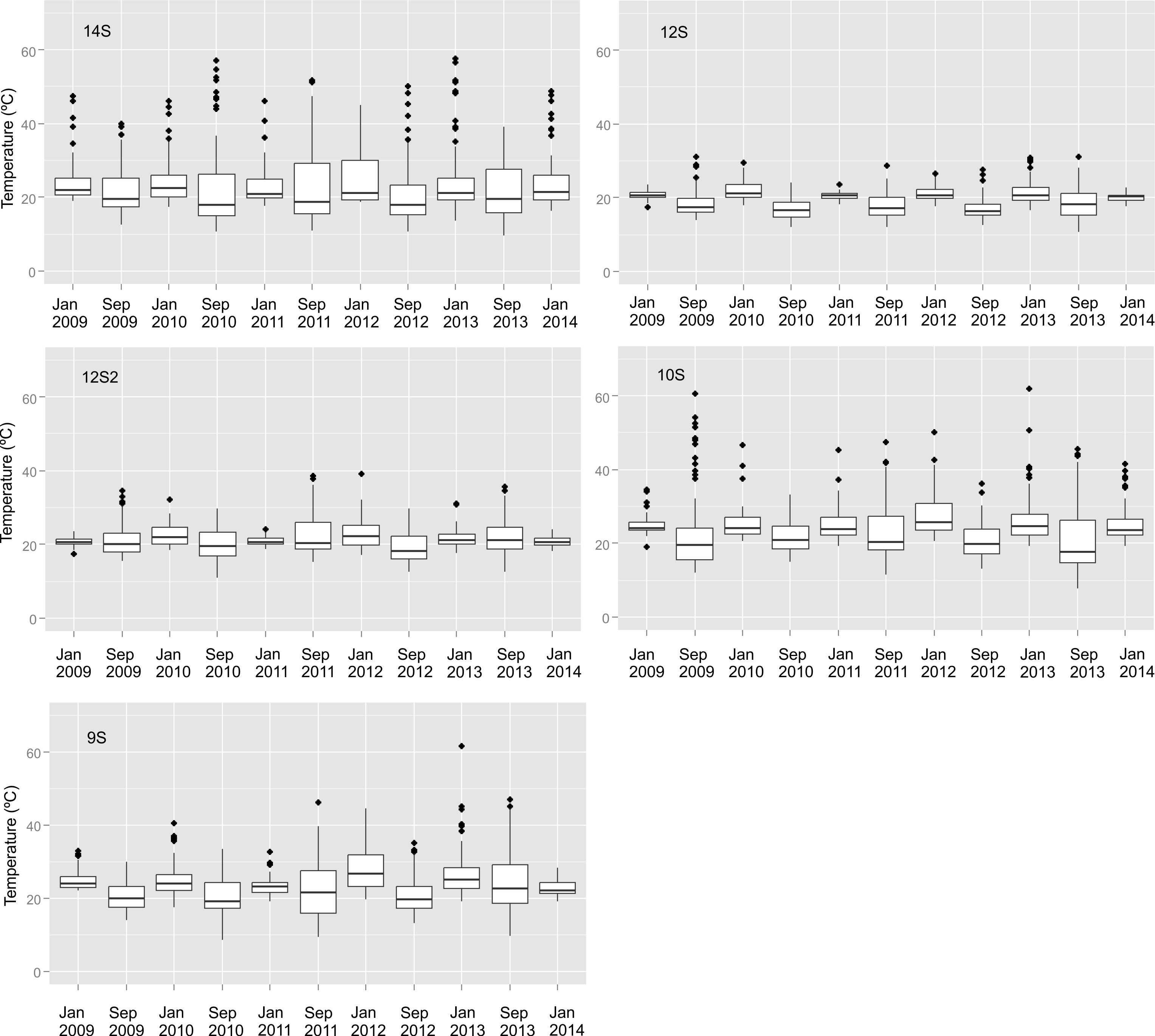

Supplement: S4 Fig — Boxplot of soil temperatures at sites during the months when ants were sampled. (TIF) [file pone.0122035.s006.tif]

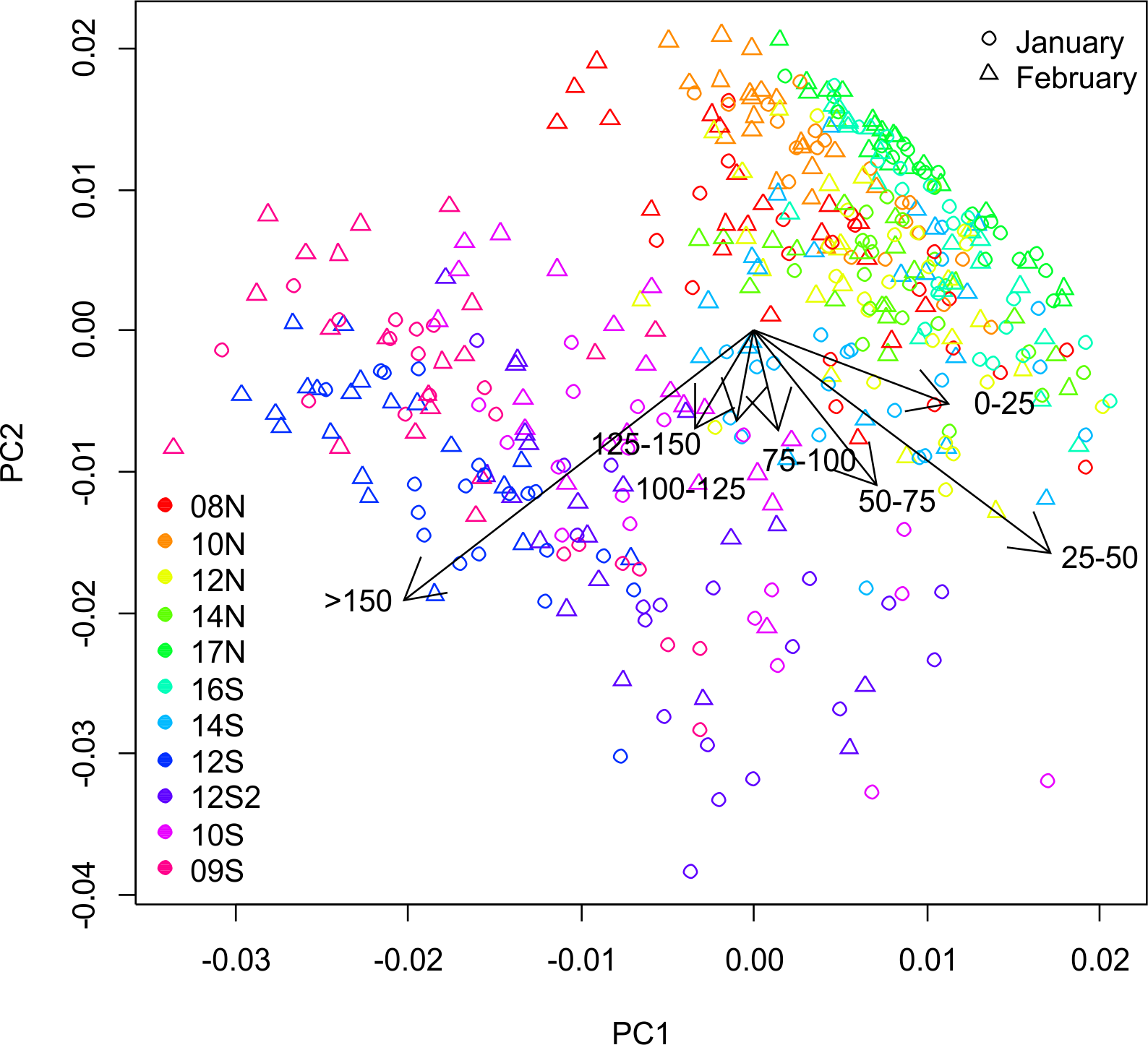

Supplement: S5 Fig — Vertical vegetation structure of all 44 replicates (averaged over the period of the study) along Soutpansberg transect. (TIF) [file pone.0122035.s007.tif]

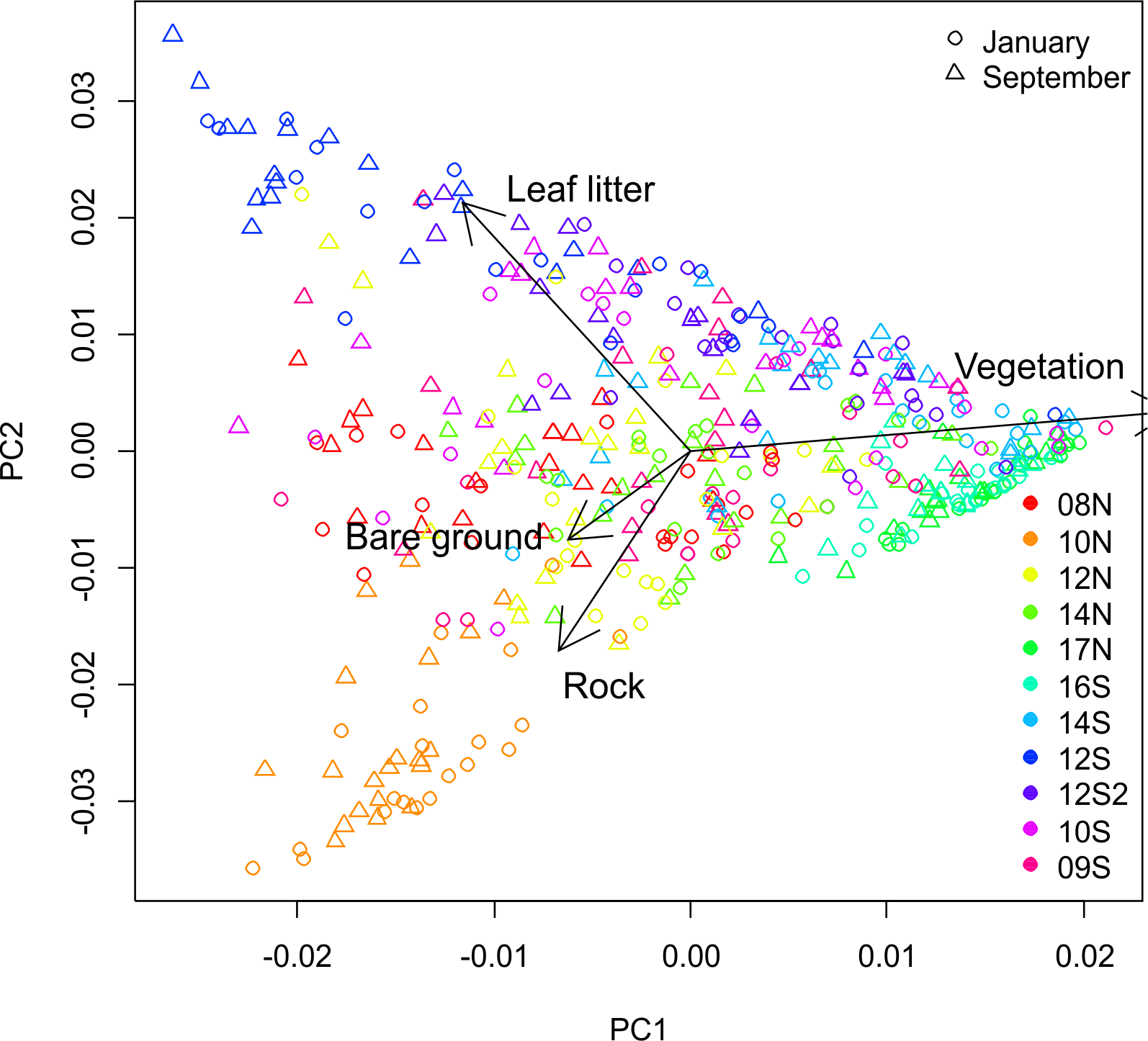

Supplement: S6 Fig — Horizontal vegetation structure of all 44 replicates (averaged over the period of the study) along Soutpansberg transect. (TIF) [file pone.0122035.s008.tif]

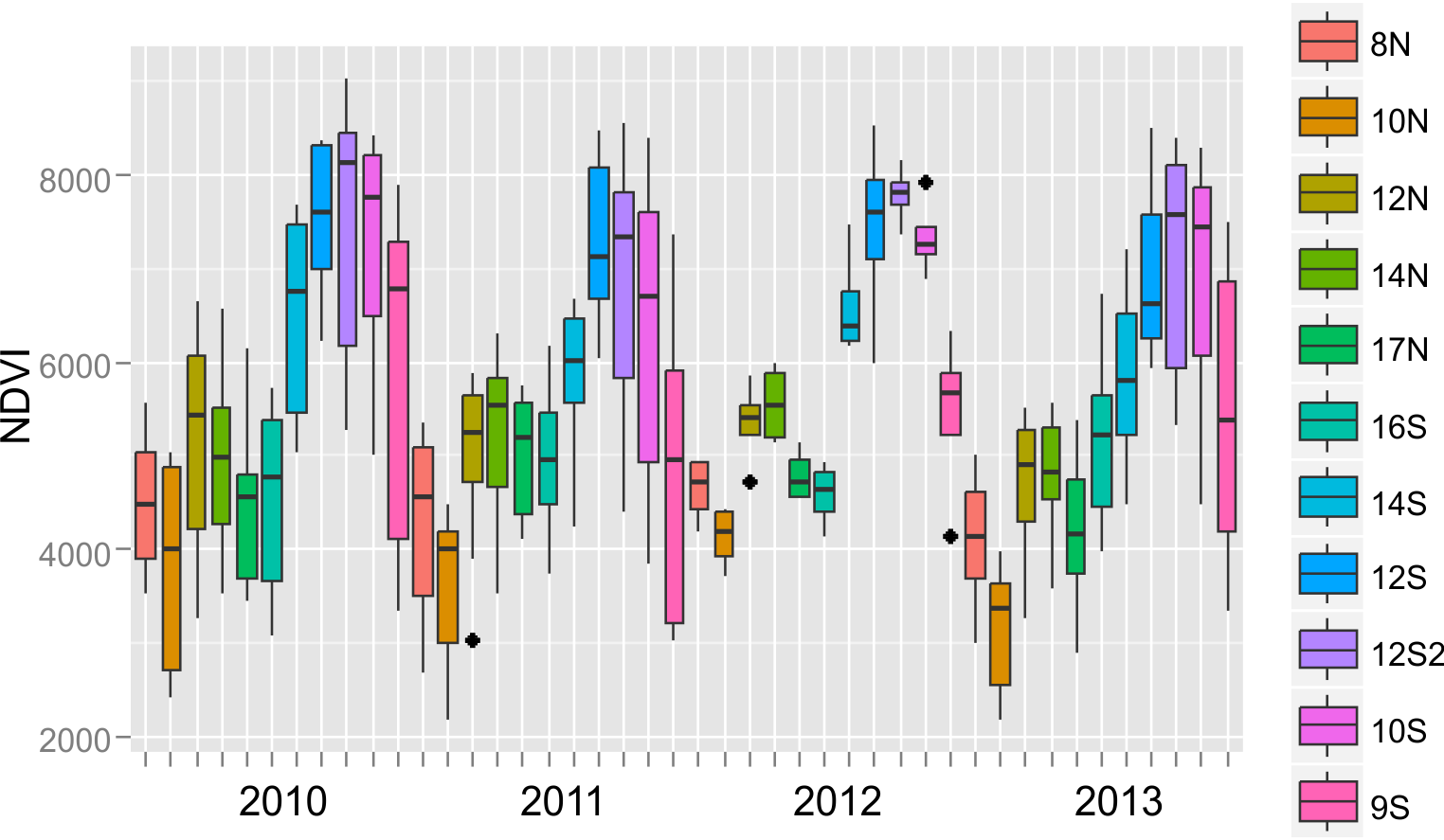

Supplement: S7 Fig — Boxplot of NDVI at each site over the period of the study. (TIF) [file pone.0122035.s009.tif]

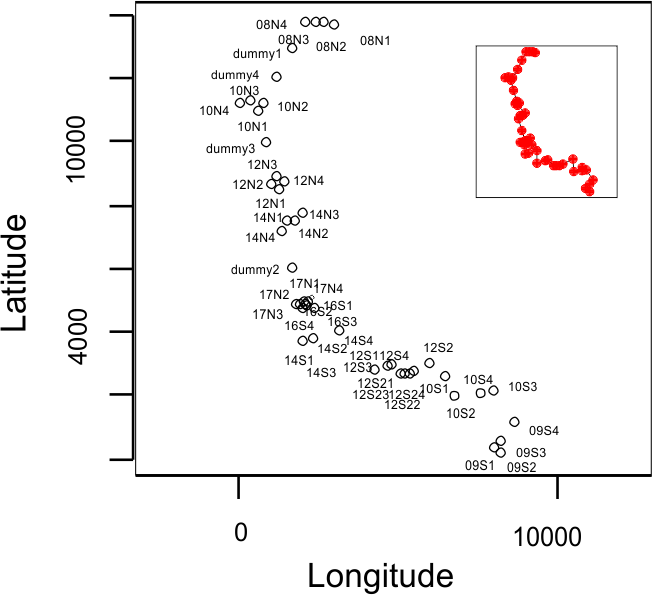

Supplement: S8 Fig — UTM coordinates of 44 replicates along transect as well as the four dummy variables included in the Principal Coordinate Analysis of Neighbourhood Matrices. Inset is the minimum spanning tree used to calculate the maximum distance required to connect all replicates. (TIF) [file pone.0122035.s010.tif]

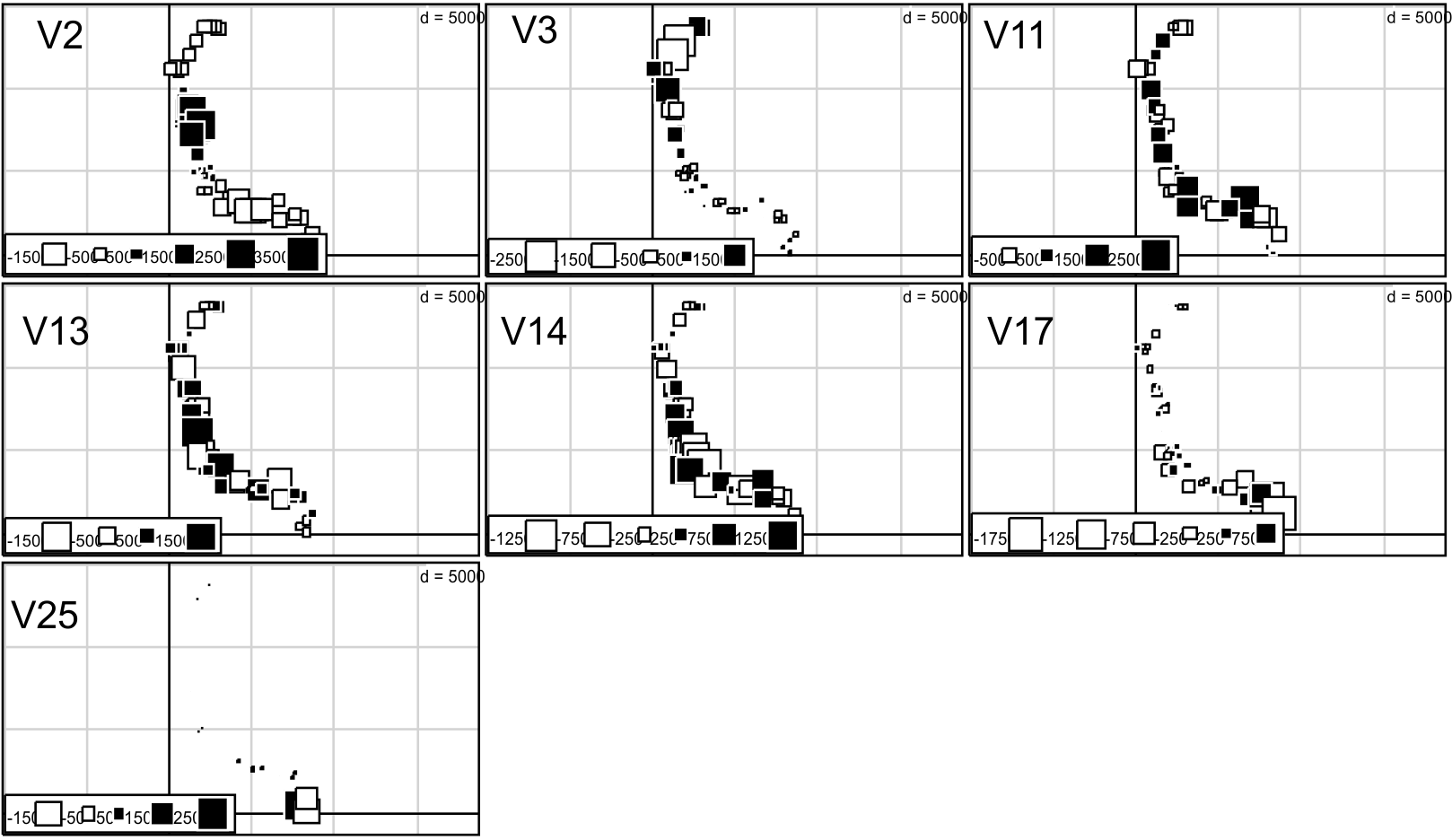

Supplement: S9 Fig — Representation of significant eigenvectors in geographical space. (TIF) [file pone.0122035.s011.tif]
