# Supplementary material for: Temporal Patterns of Ant Diversity across a Mountain with Climatically Contrasting Aspects in the Tropics of Africa
Source: PLoS One. 2015 Mar 16;10(3):e0122035. doi: 10.1371/journal.pone.0122035 (PMC4361397; doi:10.1371/journal.pone.0122035)
Supplement: S2 Table — UTM coordinates of replicates as well as dummy variables that were included to reduce the threshold value and reduce the smallest scale that can be perceived. (PDF) [file pone.0122035.s002.pdf]

Site	x	y
08N1	2939.881	13723.02
08N2	2659.836	13790.83
08N3	2353.574	13824.71
08N4	2074.313	13816.04
dummy1	1623.912	12969.59
dummy4	1173.51	12123.14
10N1	535.5868	11041.56
10N2	723.1091	11276.69
10N3	328.5784	11305.34
10N4	7.1399	11224.21
dummy3	828.9084	10010.17
12N1	1249.393	8542.018
12N2	992.5585	8705.037
12N3	1122.23	8978.785
12N4	1389.44	8822.573
14N1	1444.212	7545.258
14N2	1736.175	7584.742
14N3	1938.953	7812.952
14N4	1332.671	7251.269
dummy2	1657.551	6100.532
17N1	1743.765	4949.794
17N2	1486.017	4929.693
17N3	1982.43	5117.573
17N4	2009.877	4917.677
16S1	2239.668	5106.651
16S2	2145.626	4816.809
16S3	1864.859	4707.348
16S4	2472.614	5300.002
14S1	1926.538	3790.005
14S2	2604.859	4680.641
14S3	3104.093	4114.989
14S4	2285.512	3854.95
12S1	3913.975	3118.638
12S2	3130.374	2863.603
12S3	4191.959	3184.893
12S4	6610.348	3264.923

Site	x	y
12S21	5335.447	2642.684
12S22	5030.98	2647.81
12S23	4736.225	2620.635
12S24	5629.295	2737.454
10S1	6712.08	2066.513
10S2	7943.21	2164.164
10S3	7536.903	2150.023
10S4	7580.735	2436.264
09S1	7913.365	411.728
09S2	8299.115	64.976
09S3	8311.759	806.053
09S4	8624.829	1186.315
